# The Cure and Control of Cancer

**Published:** 1921-09-03

**Authors:** 


### THE CURE AND CONTROL OF CANCER.

THERE is little doubt that the increase occurring year by year in the incidence of cancer calls for the careful attention of the layman as well as the doctor. To some extent the tendency may be more apparent than real. The employment of new diagnostic methods, new instruments, and the development of new clinical conceptions have probably led to discovery of many cases which might formerly have passed unrecognised. Nevertheless, the most recent and reliable records suggest that there is an actual increase in the number of cancer cases, and therefore it is urgent that the people should have reliable guidance as to how they best can help the medical profession to combat it. In this particular note attention is primarily drawn to the misconceptions which have lately arisen concerning $x$-rays and the treatment of cancer, and we are requested to publish the following statement, dated August 18, which has been issued from 11 Chandos Street, W. 1, by the Council of the British Association for the Advancement of Radiology and Physiotherapy (the B.A.R.P.), a body of medical men which includes in its membership the great majority of radiologists in London and the provinces.

In view of the publicity that has been given to radiotherapy in the treatment of cancer by the publication of laudatory articles in the medical and lay Press, and the extraordinary claims that have been put forward by the authorities of the West London Hospital, it seems advisable that a considered statement on the use of these agents should be made. The treatment referred to has not yet been thoroughly tested. It possesses great potential dangers, and may not prove as efficacious as the claims now made would suggest. In the nature of the case, however, no certainty can be arrived at for some years. The unwarranted laudation of this change in technique will probably lead to a reaction and bring discredit upon $x$-ray treatment in general.

The claim put forward by the Erlangen School is that by means of their special method it is possible to administer a dose of $x$-rays which will cure cancer in one application. This claim is commented on as follows by a leader-writer in the *Lancet* of July 2, 1921:—

"It will be seen that the suggestion in the lay Press, that cases of malignant disease should go to the radiologist immediately the diagnosis is made, and before operation, is based on the observation of competent observers. There is little doubt that the time has come for us to reconsider our position in dealing with the situation."

This we regard as a most ill-advised pronouncement, and we emphatically disagree with the conclusions expressed. The time has *not* yet come when radiotherapy may be regarded as the first choice in the treatment of the majority of cases of cancer.

We believe that, of any single method, surgery still offers the best prospect of cure in nearly all cases of cancer, and that until much more convincing proof of the efficacy of $x$-rays or other form of radiation is forthcoming it would be extremely dangerous to encourage patients to trust to $x$-ray treatment alone for the cure of these very serious conditions. The possibility of successful surgical intervention ought to be, in each particular case, fully discussed.

We are, however, of the opinion that a closer co-operation between the surgeon and the radiologist would lead to a clearer appreciation of the value of radiation in treatment, and that in all cases both surgery and radiation therapy should be fully considered, with a view to making the fullest use of both. Combined treatment offers the greatest hope of success.

The methods employed in this country up to the present have given promising results. They have been worked out for use in conjunction with surgery, and it would be unwise to abandon them before we are assured that the more intensive form of treatment will give the patient an increase of favourable chances.

It should be clearly stated that radiologists in this country have, during the past few years, so far perfected their technique that the risk of any injury to the patient is now small, provided that his treatment is under the direction of a medical man of wide experience in this class of work.

The matter is receiving the most serious consideration, and the public may rest assured that if the prospects held out by the more drastic procedure prove to be better than those offered by existing methods, full advantage will be taken of it in this country.

In our opinion, the real contribution to progress on the part of the Erlangen School is that they have employed in suitable quantities $x$-rays of a higher penetration than that hitherto used, and have also carefully systematised already known methods of measuring dosage. Whether or not these rays ultimately prove superior in all cases to those of less penetration, this is an achievement for which they will always be entitled to credit.

It is unnecessary to import the apparatus from Germany; several firms in this country are now making the requisite equipment, so that difficulty of obtaining plant will not be a bar to research.

In conclusion, we wish to say once more how much it is to be regretted that hasty opinions on medical matters should be given wide publicity. Such pronouncements, unless authoritatively traversed, could not fail to be harmful to the future of radiology, in that they raise the hopes which are far from certain of realisation. $X$-rays have already relieved suffering and prolonged active life in thousands of cancer victims; they have even effected a few apparent cures, and their value in helping to prevent return after operation is now generally recognised. It would, therefore, be neither more nor less than a calamity if public disappointment resulting from unfulfilled promises were to bring discredit on radiation therapy, which is in reality a powerful agent in the warfare against disease.

